# Widely Targeted Metabolomic Analysis Revealed the Diversity in Milk from Goats, Sheep, Cows, and Buffaloes and Its Association with Flavor Profiles

**DOI:** 10.3390/foods13091365

**Published:** 2024-04-28

**Authors:** Fuhong Zhang, Yaling Wang, Baolong Liu, Ping Gong, Chenbo Shi, Lu Zhu, Jianqing Zhao, Weiwei Yao, Qingqing Liu, Jun Luo

**Affiliations:** 1 Key Laboratory of Animal Genetics, Breeding and Reproduction of Shaanxi Province, College of Animal Science and Technology, Northwest A&F University, Yangling 712100, China; zhang_fuhong@nwafu.edu.cn (F.Z.); 15637410238@nwafu.edu.cn (Y.W.); baolong@nwafu.edu.cn (B.L.); shichenbo@nwafu.edu.cn (C.S.); zhulu@nwafu.edu.cn (L.Z.); zhao2021@nwafu.edu.cn (J.Z.); yaoweiwei@nwafu.edu.cn (W.Y.); lqq@nwafu.edu.cn (Q.L.); 2 Institute of Animal Husbandry Quality Standards, Xinjiang Academy of Animal Sciences, Urumchi 830000, China; ggpp99@foxmail.com

**Keywords:** goat, milk, UPLC–ESI–MS/MS-based metabolomics, flavor profile

## Abstract

The milk flavor can be attributed to the presence of numerous flavor molecules and precursors. In this study, we employed widely targeted metabolomic analysis techniques to analyze the metabolic profiles of various milk samples obtained from goats, sheep, dairy cows, and buffaloes. A total of 631 metabolites were identified in the milk samples, which were further categorized into 16 distinct classes. Principal component analysis (PCA) suggested that the metabolite profiles of samples from the same species exhibit clustering, while separated patterns of metabolite profiles are observed across goat, sheep, cow, and buffalo species. The differential metabolites between the groups of each species were screened based on fold change and variable importance in projection (VIP) values. Five core differential metabolites were subsequently identified, including 3-(3-hydroxyphenyl)-3-hydroxypropanoic acid, inosine 5′-triphosphate, methylcysteine, N-cinnamylglycine, and small peptide (L-tyrosine–L-aspartate). Through multiple comparisons, we also screened biomarkers of each type of milk. Our metabolomic data showed significant inter-species differences in the composition and concentration of some compounds, such as organic acids, amino acids, sugars, nucleotides, and their derivatives, which may affect the overall flavor properties of the milk sample. These findings provided insights into the molecular basis underlying inter-species variations in milk flavor.

## 1. Introduction

Milk from goats, sheep, and cows show differences in composition that set the different varieties apart from each other. For instance, goat milk exhibits a higher mineral content, increased calcium levels [1], and a distinct fatty acid composition [2,3]. In addition, due to the presence of its natural antibiotics and antioxidants, goat milk is also believed to have a certain degree of immune-boosting effects [4]. Previous studies primarily focused on conducting a comparative analysis of the nutrient composition between goat milk and cow milk [5,6], as well as comparing the individual or multiple flavor-related compounds across species. However, few studies have investigated the role of metabolites in the determination of milk flavor.

Due to the intricate chemical composition of livestock products, such as milk and meats, their flavor is typically attributed not to a singular component but rather to a diverse array of molecules [7]. Previous studies have indicated that the flavor profile of milk encompasses a wide range of compounds, including free fatty acids, sulfur compounds, terpenoids, aromatic compounds, esters, ethers, aldehydes, ketones, alkanes, alcohols, and lactones [8,9]. Changes in the abundance and composition of these compounds may explain the distinct flavors observed in milk from different species. Some studies have identified branched-chain fatty acids as the primary compounds responsible for the characteristic “goaty” flavor of goat milk and sheep milk [5,10,11]. Additionally, the presence of 3-methylindole and 4-methyl phenol [12], along with oxidation byproducts of fatty acids, such as aldehydes, ketones, lactones, and stearic acid, also play a role in determining milk flavor [13].

Metabolomics provides a powerful tool for investigating intricate metabolite profiles, facilitating comprehensive investigations into the growth and development of plants and animals [14,15], stress response [16,17,18], immune interactions [19], mutant phenotypes [20,21], nutrient composition [22], bioactive compounds [23], fermentation flavor [24], brewing technology [25], etc. Recently, extensive investigations have been conducted on the correlation between microbial metabolite activity in soil, air, water, and animal intestines and human health [26,27]. Metabolomics has been employed for the analysis of food composition, identification of food quality, monitoring of food consumption, and assessment of nutrition [28]. In the field of food metabolomics, an increasing number of studies have begun to focus on the application of metabolomics in food flavor analysis. Many studies have quantified various compounds in food, including sugars, amino acids, and organic acids, to assess their contribution to the formation of food flavor [29]. For instance, the identification of major flavor compounds associated with wines [24] and radish taproots [30] has been accomplished through the application of a widely targeted metabolomics technique. The application of metabolomic analysis has also been extended to the characterization of flavor profiles in animal-derived foods. Zhang et al. used nuclear magnetic resonance (NMR) to investigate metabolites of dry-cured hams, revealing that amino acids and organic acids played a predominant role in determining the taste profile [31]. Based on liquid chromatography mass spectrometry (LC-MS) techniques, Wang et al. compared metabolites in three types of goat meat and revealed that fatty acids, aldehydes, ketones, lactones, alkaloids, flavonoids, and phenolics were responsible for the nuances of their flavors [32].

Widely targeted metabolomics represents an advancement technique in the field of metabolomics, integrating the merits of both targeted and untargeted methods. It offers remarkable advantages, such as high throughput, enhanced sensitivity, and extensive coverage [33,34]. By utilizing a self-constructed compound database and employing the multiple reaction monitoring (MRM) scanning mode of mass spectrometry, this method facilitates both qualitative and quantitative identification of over a thousand metabolites. This tool enables us to investigate the metabolic profiles of milk from different species and the identification of biomarkers associated with milk flavor.

In this work, we employed a widely targeted metabolomic mean to compare the composition and relative abundance of milk metabolites across multiple species, including goats, sheep, cows, and buffaloes. Notably, characteristic metabolites specific to each type of milk and their associated metabolic pathways were identified. The findings are anticipated to contribute to a more comprehensive understanding of the regulation of milk flavor and propose further research for manipulating the flavor of milk products.

## 2. Materials and Methods

### 2.1. Milk Samples

Xinong Saanen dairy goats (approximately 3–4 years old, 2 parities) and Holstein cows (approximately 4–5 years old, 2 parities) utilized in this study were selected from the experimental farm located at Northwest A&F University, Yangling, Shaanxi Province, China. The East Friesian dairy sheep (approximately 3 to 4 years old, 2 parities) utilized in this research were selected from Yuan Sheng Nong Mu Co., Ltd., Jinchang, Gansu Province, China. The buffaloes (approximately 4 to 5 years old, 2 parities) utilized in this study were selected from a commercial farm located in Guangxi, Province, China. The animals were all managed similarly and were provided with a mixed diet consisting of corn, soybean meal, bran, rapeseed meal, and a mineral-vitamin premix. The milk samples were collected from each dairy animal during the peak lactation period (60 days postpartum; 6 goats, GMM group; 6 sheep, SMM group; 6 cows, CMM group; 6 buffaloes, BMM) (Table 1), with a sample volume of 100 mL. Subsequently, the collected samples were divided into centrifuge tubes of 50 mL capacity, ensuring secure seals. Finally, the samples were stored at −80 °C in a refrigerator.

### 2.2. Sample Preparation and Extraction

The samples stored at −80 °C were thawed on ice until there was no ice in the sample and vortexed for 10 s. Subsequently, 50 μL of the sample and 300 μL of extraction solution (ACN:methanol = 1:4, *v*/*v*) containing internal standards were added to a 2 mL microcentrifuge tube. The sample was vortexed for 3 min and then centrifuged at 12,000 rpm for 10 min (at a temperature of 4 °C). A volume of 200 μL of the supernatant was collected and placed in a freezer set at −20 °C for a duration of 30 min, followed by centrifugation at 12,000 rpm for another period of three minutes (at a temperature of 4 °C). An aliquot consisting of 180 μL from the supernatant was transferred for LC-MS analysis. The sample extracts were analyzed using ultra-performance liquid chromatography (UPLC, ExionLC AD (AB SCIEX Pet. Ltd., Framingham, MA, USA), https://sciex.com.cn/, accessed on 1 December 2022) and tandem mass spectrometry (MS/MS, QTRAP^®^ (AB SCIEX Pet. Ltd., Framingham, MA, USA), https://sciex.com/, accessed on 1 December 2022).

### 2.3. Ultra-Performance Liquid Chromatography Conditions

The Ultra-Performance Liquid Chromatography (UPLC) conditions were as follows: the chromatographic column used was Waters ACQUITY UPLC HSS T3 C18, with a particle size of 1.8 µm and dimensions of 2.1 mm × 100 mm; the mobile phase consisted of ultra-pure water (supplemented with 0.1% formic acid) as phase A and acetonitrile (supplemented with 0.1% formic acid) as phase B; the gradient program employed was Water/Acetonitrile, starting at a ratio of 95:5 *v*/*v* at 0 min, transitioning to a ratio of 10:90 *v*/*v* at 11.0 min, maintaining this ratio until 12.0 min, then returning to a ratio of 95:5 *v*/*v* at 12.1 min and continuing until the endpoint at 14.0 min; the flow rate utilized was set at a constant value of 0.4 mL/min; and the column temperature was maintained at a steady level of 40 °C throughout analysis.

### 2.4. Tandem Mass Spectrometry Conditions

LIT and triple quadrupole (QQQ) scans were acquired on a triple quadrupole-linear ion trap mass spectrometer (QTRAP), QTRAP^®^ LC-MS/MS System (AB Sciex, Shanghai, China), equipped with an ESI Turbo Ion-Spray interface (AB SCIEX Pet. Ltd., Framingham, MA, USA) operating in positive and negative ion mode and controlled by Analyst 1.6.3 software (Sciex, AB SCIEX Pet. Ltd., Framingham, MA, USA). Electrospray ionization (ESI) was performed at a temperature of 500 °C, with the mass spectrum voltage set to 5500 V (positive) and −4500 V (negative). The ion source gas I (GSI) pressure was maintained at 55 psi, while the gas II (GS II) pressure was set to 60 psi. Additionally, the curtain gas (CUR) pressure was adjusted to 25 psi. For collision-activated dissociation (CAD), the parameter set was optimized for high performance. In a triple quadrupole (Qtrap), each ion pair underwent scanning based on an optimized declustering potential (DP) and collision energy (CE).

### 2.5. Unsupervised Principal Component Analysis

The principal component analysis (PCA) was conducted using the statistical function prcomp in R (www.r-project.org, accessed on 1 May 2023), with the parameter “scale” set to “True”. Unit variance scaling is calculated by centralizing the raw data and dividing it by the standard deviation of the variable. The calculating formula is as follows:

$$x^{\prime }=\frac{x-u}{\sigma }$$

Here, u is the mean, and σ is the standard deviation.

### 2.6. Orthogonal Partial Least Squares-Discriminant Analysis (OPLS-DA)

The partial least squares-discriminant analysis (PLS-DA) is a supervised multivariate statistical analysis method employed for pattern recognition. This specific approach involves extracting the components of both the independent variable X and dependent variable Y, followed by calculating their correlation. OPLS-DA integrates orthogonal signal correction (OSC) and PLS-DA methods to decompose the X matrix information into two components, one related to Y and the other unrelated to Y, effectively capturing relevant variance while removing irrelevant differences [35]. OPLS-DA further applies centralized processing after the log2 transformation of the original data. Here, X represents the matrix of sample quantitative information, while Y represents the matrix of sample grouping information.

The prediction parameters of the OPLS-DA evaluation model encompass R^2^X, R^2^Y, and Q^2^. Herein, R^2^X and R^2^Y denote the explanatory power of the constructed model for X and Y matrices respectively, while Q^2^ represents the predictive capability of the model. The closer these three indicators approach 1, the greater stability and reliability exhibited by the model. A Q^2^ value greater than 0.5 indicates an effective model, while a Q^2^ value exceeding 0.9 signifies an excellent model.

### 2.7. Differential Metabolites Selected

The differential metabolites for different types of milk were identified based on the criteria of VIP ≥ 1 and |Log2FC| ≥ 1.0. VIP values are extracted from OPLS-DA results by using the R software package “MetaboAnalystR”, which includes score plots and permutation plots. The data underwent logarithmic transformation (log2) and mean centering before OPLS-DA. To avoid overfitting, we performed a permutation test (200 permutations).

### 2.8. Kyoto Encyclopedia of Genes and Genomes (KEGG) Annotation and Enrichment Analysis

The identified metabolites were annotated using the KEGG Compound database (http://www.kegg.jp/kegg/compound/, accessed on 1 May 2023) and subsequently mapped to the KEGG Pathway database (http://www.kegg.jp/kegg/pathway.html, accessed on 1 May 2023). The identified pathways, which exhibited significant regulation of metabolites, were subsequently subjected to metabolite set enrichment analysis (MSEA) using a hypergeometric test to determine their statistical significance. The calculating formula is as follows:

$$P=1-\sum_{i=0}^{m-1}\frac{\left(\begin{array}{c} M\\ i \end{array}\right)\left(\begin{array}{cc} N & -M\\ n & -i \end{array}\right)}{\left(\begin{array}{c} N\\ n \end{array}\right)}$$

Among these, N represents the total number of metabolites annotated in the KEGG database, n represents the count of differential metabolites among all annotated metabolites, M denotes the overall number of metabolites within a specific KEGG pathway, and m signifies the count of differential metabolites within the pathway.

## 3. Results

### 3.1. Metabolite Profiles of Milk Derived from Goat, Sheep, Cow, and Buffalo Species

**Figure 1:**
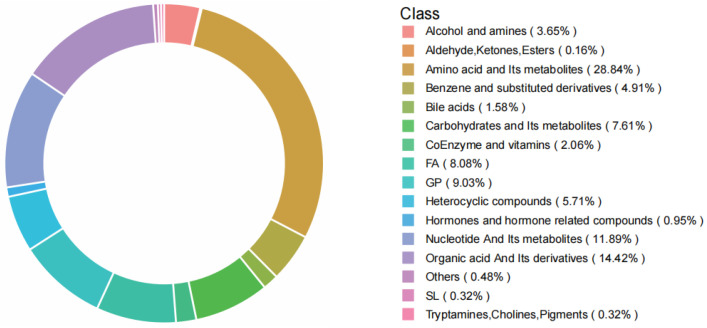
The proportion of different classes of compounds for the metabolites detected in milk samples obtained from goats, sheep, cows, and buffaloes using a widely targeted metabolomic approach.

To gain deeper insights into the metabolite variations among milk from diverse species, we conducted the widely targeted UPLC-MS/MS metabolomic analysis on a total of 24 distinct milk samples. A total of 631 metabolites were identified and classified into 16 categories (Table S1), including 182 amino acids and their derivatives (28.84%), 91 organic acid and their derivatives (14.42%), 75 nucleotides and derivatives (11.82%), 57 glycerophospholipids (GP, 9.03%), 51 fatty acyls (FA, 8.08%), 48 carbohydrates and its metabolites (7.61%), 36 heterocyclic compounds (5.71%), 31 benzene and substituted derivatives (4.91%), 23 alcohol and amines (3.65%), 13 coenzyme and vitamins (2.06%), 10 bile acids (1.58%), 6 hormones and hormone-related compounds (0.95%), 2 sphingolipids (SL, 0.32%), 2 tryptamines, cholines, and pigments (0.32%), and others (Figure 1).

**Figure 2:**
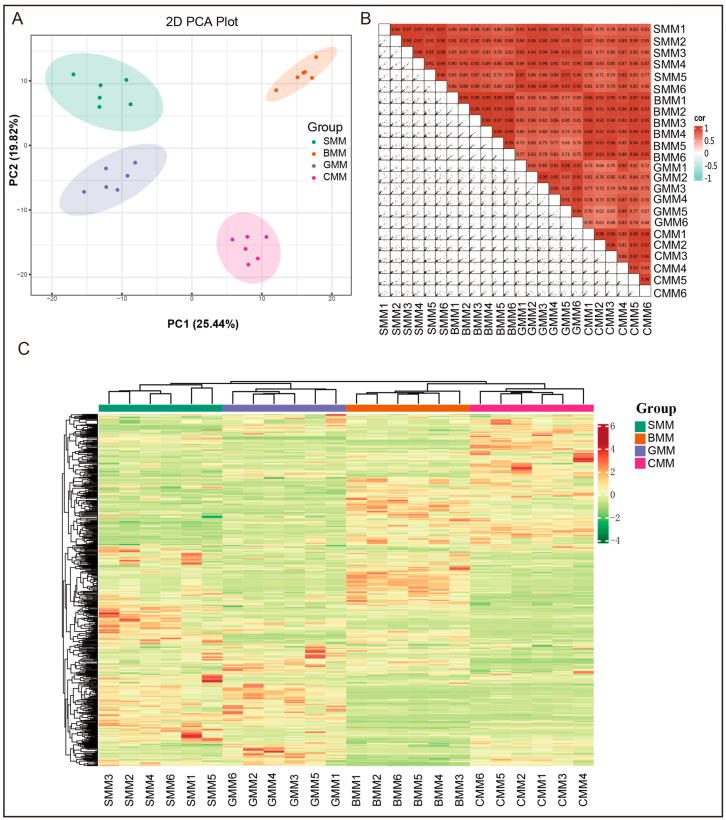
Principal component analysis (PCA), correlation analysis, and cluster analysis were conducted to understand the overall metabolite differences between the groups of milk samples and the variation between the samples within the groups. (**A**) PCA plot showing the different metabolic profiles of milk samples. (**B**) Clustering heat map showing the Pearson correlation coefficients of metabolites in 24 milk samples. (**C**) The cluster heat map displaying the accumulation levels of each metabolite across four different types of milk. SMM1-6, the samples of sheep milk metabolites. BMM1-6, the samples of buffalo milk metabolites. GMM1-6, the samples of goat milk metabolites. CMM1-6, the samples of cow milk metabolites.

The principal component analysis (PCA) [36] was conducted using the prcomp function in R (www.r-project.org, accessed on 1 May 2023). We observed that the metabolite compositions of the four types of milk exhibited clear separation, with PC1 and PC2 explaining 25.44% and 19.82% of the total variance, respectively (Figure 2A). The PCA plot also demonstrated that the quality control (QC) samples, prepared from a mixture of samples, exhibited clustering within the same region and even some overlap, thereby indicating their similarity in metabolic profiles and affirming the stability and reproducibility of our analysis (Figure S1).

Pearson correlation coefficients between samples were calculated using the cor function in R. The results depicted in Figure 2B demonstrate a strong correlation coefficient among the samples, indicating the robust reproducibility of our analysis and providing a high level of confidence in discerning differences between milk samples from distinct species (Table S2). A hierarchical cluster analysis was performed after the data was processed by unit variance scaling. The cluster heat map was drawn by using the R program script. It was observed that milk samples of the same type exhibited clustering tendencies (Figure 2C). These findings indicated that there are significant differences in metabolite profiles between the four types of milk.

**Figure 3:**
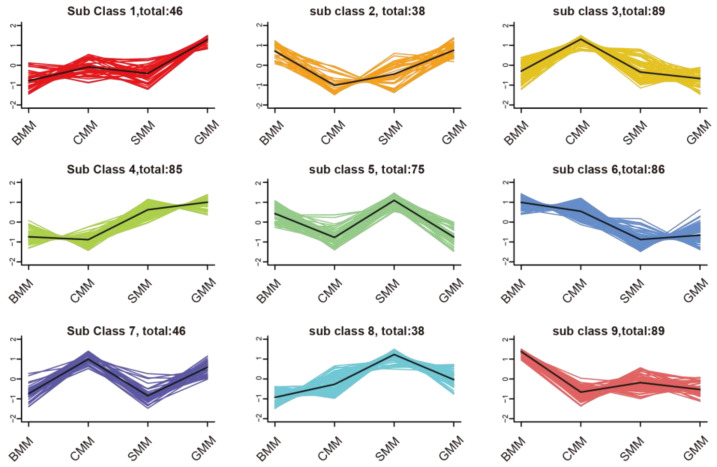
K-Means analysis. The X-axis represents the sample groups, while the Y-axis denotes the standardized relative content of metabolites. Additionally, sub-class indicates metabolites exhibiting similar trends.

To investigate the trend of relative abundance changes in metabolites from BMM, CMM, and SMM to GMM, we conducted a K-Means cluster analysis on the standardized data of all metabolites. The result showed that the metabolites were classified into nine subclasses based on the trend of relative abundance changes (Figure 3 and Table S3). The number of metabolites in subclasses 3 and 9 reached the highest count, totaling 89 for both subclasses, while subclass 2 exhibited the lowest enrichment of metabolites, with a total of only 38. In subclasses 1 and 4, the GMM group showed higher metabolite abundance levels compared to the other three groups. In subclass 3, the CMM group exhibited elevated metabolite abundance levels in comparison to the other three groups. In subclass 8, the SMM group displayed higher relative abundance levels of metabolites when compared to the remaining three groups. In subclasses 6 and 9, the BMM group demonstrated higher relative abundance levels of metabolites than the other three groups. These up-regulated metabolites may play a role in determining the distinct flavor profiles of the various types of milk (Figure 3).

### 3.2. Differential Metabolite Screening

**Figure 4:**
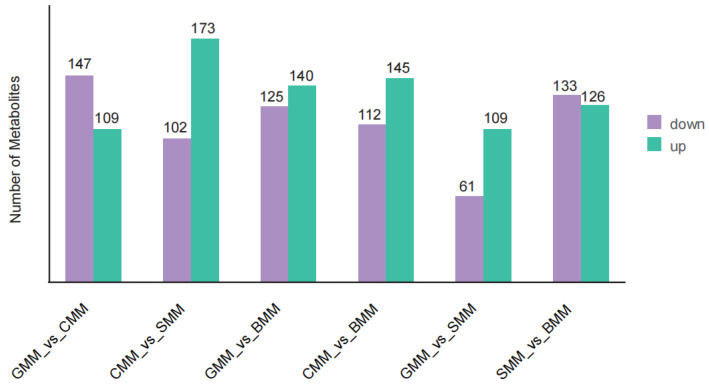
Histogram of the differential metabolites. The numbers of up-regulated (represented in green) and down-regulated (represented in purple) metabolites between each pair of experimental groups.

An OPLS-DA [37] analysis was employed to accurately identify the differential metabolites in the comparison groups. The Q^2^ values of all comparison groups exceeded 0.9, indicating the robustness and reliability of these models for further screening of differential metabolites (Figure S2). Then, we integrated the fold change with VIP values derived from the OPLS-DA model for the identification of differentially expressed metabolites (DEMs). The DEMs were identified based on a threshold of VIP ≥ 1.0, with fold changes ≥2 and ≤0.5. We obtained six DEM sets, including GMM vs. CMM, GMM vs. SMM, GMM vs. BMM, CMM vs. SMM, CMM vs. BMM, and SMM vs. BMM. There were 256 DEMs (109 up-regulated and 147 down-regulated) in the GMM vs. CMM, 170 DEMs (109 up-regulated and 61 down-regulated) in the GMM vs. SMM, 265 DEMs (140 up-regulated and 125 down-regulated) in GMM vs. BMM, 257 DEMs (145 up-regulated and 112 down-regulated) in CMM vs. BMM, 275 DEMs (173 up-regulated and 102 down-regulated) in CMM vs. SMM, and 259 DEMs (126 up-regulated and 133 down-regulated) in SMM vs. BMM (Figure 4 and Tables S4–S9). The results revealed that the dissimilarities in the number of differential metabolites between goat milk and sheep milk were comparatively less than those observed between goat milk and cow milk, as well as buffalo milk.

### 3.3. KEGG Enrichment Analysis of Differential Metabolites

**Figure 5:**
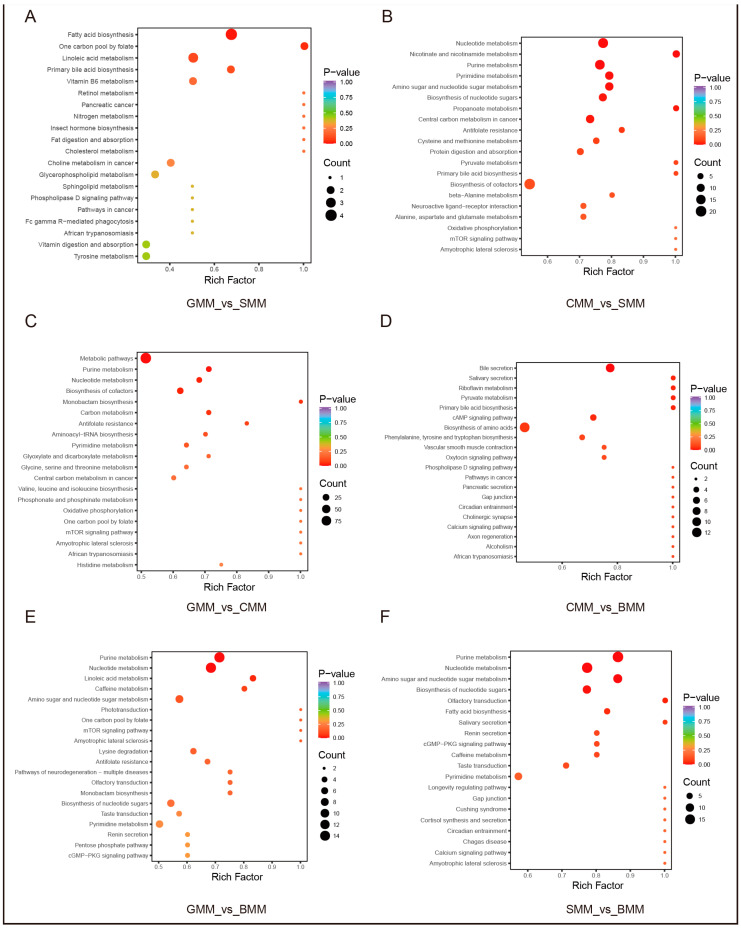
KEGG enrichment analysis of the differential metabolites. (**A**) The top 20 metabolic pathways with the lowest *q*-values in the GMM/SMM set. (**B**) The top 20 metabolic pathways with the lowest *q*-values in the CMM/SMM set. (**C**) The top 20 metabolic pathways with the lowest *q*-values in the GMM/CMM set. (**D**) The top 20 metabolic pathways with the lowest *q*-values in the CMM/BMM set. (**E**) The top 20 metabolic pathways with the lowest *q*-values in the GMM/BMM set. (**F**) The top 20 metabolic pathways with the lowest *q*-values in the SMM/BMM set.

To obtain comprehensive insights into the metabolic pathways of differential metabolites, we performed KEGG enrichment analysis on 460 differential metabolites among four species (Figure 5A–F). The enrichment analysis revealed that the differential metabolites in the GMM/CMM and GMM/BMM groups were primarily associated with purine metabolism and nucleotide metabolism (Figure 5C,E). Moreover, these two metabolic pathways also showed up in the SMM/CMM and SMM/BMM groups (Figure 5B,F). The differential metabolites in the GMM/SMM set were mainly involved in fatty acid biosynthesis, one carbon pool by folate, and linoleic acid metabolism (Figure 5A). The differential metabolites in the CMM/BMM set showed significant enrichment in bile secretion, salivary secretion, and riboflavin metabolism (Figure 5D). The findings suggest that these pathways may play a critical role in modulating flavor characteristics and have significant biological implications.

### 3.4. Identification of Characteristic Metabolites of Each Type of Milk

**Figure 6:**
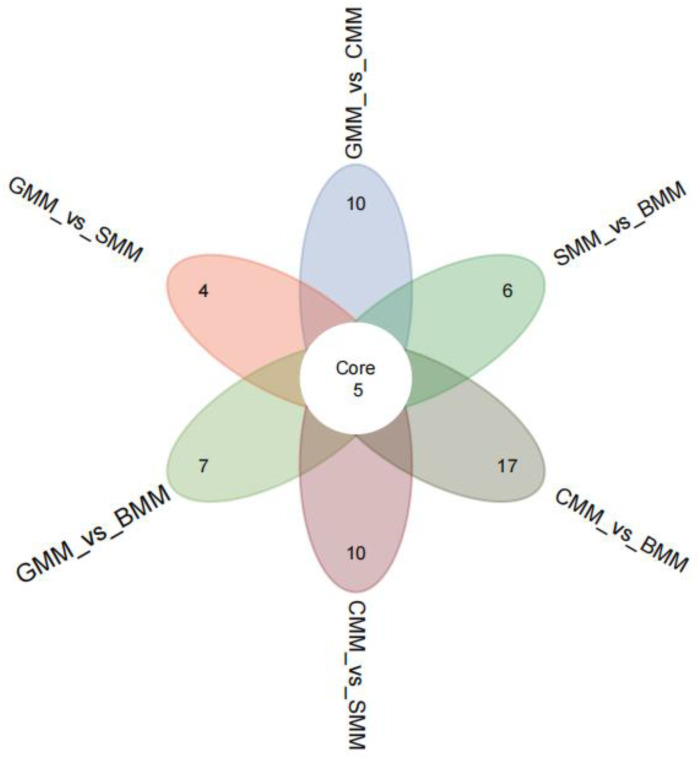
Venn diagram of differential metabolites for each comparison group of GMM, SMM, CMM, and BMM.

We conducted a comparative analysis of the DEMs in six DEM sets and screened five core differential metabolites, namely 3-(3-hydroxyphenyl)-3-hydroxypropanoic acid, inosine 5′-triphosphate, methylcysteine, N-cinnamylglycine, and Tyr-Asn (Figure 6). The findings imply that these compounds could potentially account for the variations in milk flavor observed among the species.

**Figure 7:**
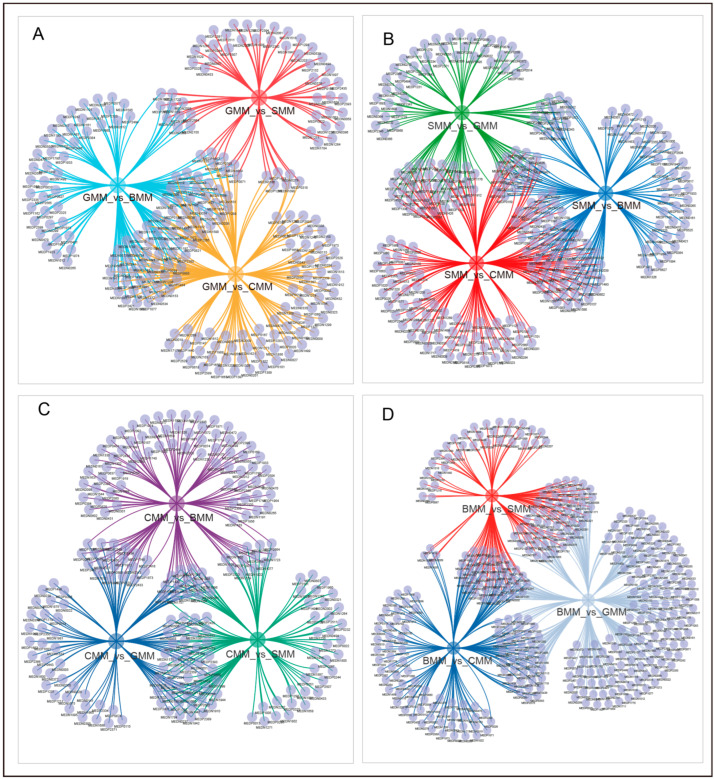
Network Venn diagram showing the characteristic metabolites of each type of milk. (**A**) The characteristic metabolites of goats. (**B**) The characteristic metabolites of sheep. (**C**) The characteristic metabolites of cows. (**D**) The characteristic metabolites of buffaloes.

To identify the characteristic metabolites of goat milk, we performed multiple comparisons of up-regulated DEMs among the GMM/CMM, GMM/SMM, and GMM/BMM sets. A total of seven characteristic metabolites were identified in goat milk, including N-(3-indolylacetyl)-L-alanine, pyridoxine 5′-phosphate, ADP-ribose, N-acetytryptophan, 3-methylcrotonyl glycine, dihydro-D-sphingosine, and N-cinnamylglycine (Figure 7A). These compounds were enriched in goat milk compared to other types of milk, suggesting their potential as biomarkers for screening goat milk.

Based on the multiple comparisons of up-regulated DEMs among the SMM/CMM, SMM/GMM, and SMM/BMM sets, a total of 18 characteristic metabolites were identified (Figure 7B). These included eight small peptides, five nucleotides and their metabolites, one amino acid, one amino derivative, one amine, one organic acid derivative, and one heterocyclic compound. The relatively elevated levels of methylcysteine, adenine, 5′-deoxy-5′-(methylthio) adenosine, N-alpha-acetyl-L-asparagine, cytidine 2′, 3′-cyclomonophosphate, oxypurinol, 8-Azaguanine, 1,4-Dihydro-1-Methyl-4-Oxo-3-Pyridinecarboxamide, 3-(3-Hydroxyphenyl)-3-hydroxypropanoic acid, and N(alpha)-acetyl-epsilon-(2-propenal) lysine, in comparison to other metabolites, can serve as potential biomarkers of sheep milk.

By conducting multiple comparisons of up-regulated DEMs among the CMM/GMM, CMM/SMM, and CMM/BMM sets, we observed 14 characteristic metabolites in cow milk. These included five carbohydrates and their metabolites, three nucleotides and their metabolites, two amino acids and their derivatives, two organic acid and their derivatives, one coenzyme and vitamin, and one fatty acyl (Figure 7C). The levels of these 14 metabolites were found to be significantly elevated in cow milk, indicating their potential as biomarkers for the screening of cow milk.

A total of 38 metabolites were identified as characteristic metabolites in buffalo milk through multiple comparisons of up-regulated DEMs among the BMM/GMM, BMM/SMM, and BMM/CMM sets. These included thirty glycerophospholipids, three amino acids and their metabolites, two fatty acyls, one hormone, one carbohydrate and its metabolite, and one organic acid (Figure 7D). We found that glycerophospholipids are the most important characteristic metabolites in buffalo milk, especially the relatively high levels of lysophosphatidylcholine (LPC), including PC (O-16:0/O-1:0), LPC (16:0/0:0), LPC (0:0/16:0), LPC (O-16:0/2:0), LPC (18:0/0:0), and LPC (0:0/18:0).

## 4. Discussion

The milk derived from goats, sheep, cows, and buffaloes serves as a significant protein source for human consumption. Previous research has primarily focused on conducting comparative analyses of the nutrient composition between goat milk and cow milk [6], as well as identifying individual or multiple compounds that contribute to their flavors [10,38]. The current knowledge regarding the global metabolic profiles among different dairy animals is limited. In this study, we focused on the overall differences in metabolic profiles among different milk types obtained from distinct species. Furthermore, we conducted a comprehensive and in-depth analysis of the flavor profiles and metabolic characteristics of four milk types while also screening their metabolic markers specific to each type.

By conducting a principal component analysis (PCA) and a cluster analysis on the population sample, we found that the metabolite profiles of milk from goats and sheep were more similar than those of milk from cows and buffaloes. The different metabolites between goat milk and milk from cow and buffalo are mainly concentrated in amino acids and their derivatives, nucleotides, and their metabolites, as well as organic acids and their derivatives. This difference also exists in sheep milk. The difference in metabolites between goat milk and sheep milk is mainly reflected in the components of amino acids and their derivatives. KEGG pathway enrichment analysis showed that the differential metabolites of goat milk were significantly enriched into two pathways, namely purine metabolism and nucleotide metabolism, compared with cow milk and buffalo milk. Compared with cow milk and buffalo milk, the differential metabolites of sheep milk were significantly enriched in three pathways, namely purine metabolism, nucleotide metabolism, amino sugar metabolism, and nucleotide sugar metabolism. The shared pathways of goat and sheep milk mainly include purine metabolism and nucleotide metabolism. The findings provide novel perspectives for comprehending the variations in metabolic profiles among diverse milk types derived from distinct species.

The presence of organic acids, amino acids, and nucleotides plays an important role in determining the flavor characteristics [10,39,40]. In this study, our results identified five core differential metabolites in four types of milk, including 3-(3-hydroxyphenyl)-3-hydroxypropanoic acid, inosine 5′-triphosphate, methylcysteine, N-cinnamylglycine, and small peptide (Tyr-Asn). Previous studies have revealed that the abnormal 3-(3-hydroxyphenyl)-3-hydroxypropanoic acid concentrations in the body are correlated with dysregulation of the intestinal microbiota and a variety of neurological diseases [41]. Inosine 5′-triphosphate (ITP) can support the initiation of effector systems [42]. In addition, the interaction of proteins and fats with volatile flavor compounds also affects humans’ perception of flavor [43,44]. Our metabolomic analysis revealed distinct profiles of lipids, as well as organic acids and their derivatives in goat milk compared to that of cows and buffaloes. Specifically, the levels of FAs (carnitine C6:0, carnitine C8:0, carnitine C7:0, and carnitine C4: DC) and short-chain fatty acids and their derivatives (methylmalonic acid, tricarballylic acid, 5-hydroxyhexanoic acid, 2-hydroxyisocaproic acid, and 8-aminooctanoic acid) in goat milk were significantly higher than those found in other species. Sheep milk exhibited significantly higher levels of FAs (carnitine C6:0, carnitine C16:0, carnitine C7:0), as well as short-chain fatty acids and their derivatives (3-(3-hydroxyphenyl)-3-hydroxypropanoic acid, tricarballylic acid, and glycerophosphoric acid) than other type milk. We also found variations in the composition and concentration of sugars and organic acids in four types of milk, which may be another factor in the distinctive flavor profiles [45]. Taken together, the differential profiles of organic acids, amino acids, and nucleotides across the four types of milk could potentially contribute to their flavor variations.

The present study reveals a wider range of metabolites in milk compared to the previous investigation and encompasses a higher multitude of metabolites. A total of 631 distinct metabolites were detected across various milk varieties. Our metabolomic data have revealed significant inter-breed variations in the compositions and concentrations of organic acids, amino acids, sugars, and nucleotides, which potentially contribute to the overall flavor attributes of milk samples. We observed that methylmalonic acid was relatively more abundant in goat milk compared to sheep milk, cow milk, and dairy milk. The activation of methylmalonic acid by gene *ACSF3* (Acyl-CoA synthetase family member 3) leads to the production of methyl malonyl-CoA, which serves as a precursor for the synthesis of methyl-branched fatty acids [46]. Some studies have identified branched-chain fatty acids as the primary compounds responsible for the characteristic “goaty” flavor of goat milk [10]. The main source of methylmalonic acid is the metabolism of propionic acid and the catabolism of branched-chain amino acids, indicating that these pathways may play a significant role in the development of characteristic flavor in goat milk. Additionally, the main sugar in milk is lactose, which is slightly lower in goat milk than in cow milk. However, goat milk contains high levels of oligosaccharides and sugar complexes [47], aligning with our findings. These compounds play important roles in various biological processes, such as cell signaling and energy metabolism [48]. The high concentration of these sugars in goat milk may have implications for human health. The levels of UDP-sugars, such as UDP-glucose, UDP-galactose, and UDP-xylose, were found to be higher in goat milk compared to other types of milk in this study.

Despite being an emerging omics technique with the ability to qualitatively and quantitatively analyze a wide range of low molecular weight metabolites in biological samples, widely targeted metabolomics is still in its nascent stage and encounters several challenges. For example, there is no single technique that can analyze all the compounds in the metabolome at the same time. In this study, we exclusively identified one ester (ethyl hydrogen malonate), while no aldehydes and ketones were detected, despite their known association with flavor [49]. In future studies, this can be solved using selective extraction techniques combined with parallel analysis of various analysis techniques. In addition, milk proteins are also important factors influencing flavor production and release [50,51], and we will further integrate metabolomics and proteomics to systemically reveal flavor markers in four types of milk.

## 5. Conclusions

Using widely targeted metabolomic technology, the metabolic profiles of goat milk, sheep milk, cow milk, and buffalo milk were systematically compared. A total of 631 metabolites were identified and classified into 16 categories. Among these, amino acids and their derivatives accounted for the highest proportion (28.84%), followed by organic acids and their derivatives (14.42%). Principal component analysis and hierarchical cluster analysis revealed that the metabolites of goat milk and sheep milk exhibited similar characteristics. Five metabolites, including 3-(3-hydroxyphenyl)-3-hydroxypropanoic acid, inosine 5′-triphosphate, methylcysteine, N-cinnamylglycine, and small peptide (Tyr-Asn), were core differential metabolites in four types of milk. The biomarkers for each type of milk were obtained through a systematic comparison of the metabolic profiles derived from goats, sheep, cows, and buffaloes. Our metabolomic data have revealed significant inter-breed variations in the compositions and concentrations of organic acids, amino acids, sugars, and nucleotides. These differences could potentially contribute to the overall flavor attributes of milk samples. The present findings are expected to contribute to a more comprehensive understanding of the regulation of milk flavor and to support further research on manipulating the flavor of milk products.

## Figures and Tables

**Table 1:** Sample information.

Species	Type of Samples	Name of Samples	Group
Goat	Milk	GMM1	GMM
Goat	Milk	GMM2	GMM
Goat	Milk	GMM3	GMM
Goat	Milk	GMM4	GMM
Goat	Milk	GMM5	GMM
Goat	Milk	GMM6	GMM
Sheep	Milk	SMM1	SMM
Sheep	Milk	SMM2	SMM
Sheep	Milk	SMM3	SMM
Sheep	Milk	SMM4	SMM
Sheep	Milk	SMM5	SMM
Sheep	Milk	SMM6	SMM
Cow	Milk	CMM1	CMM
Cow	Milk	CMM2	CMM
Cow	Milk	CMM3	CMM
Cow	Milk	CMM4	CMM
Cow	Milk	CMM5	CMM
Cow	Milk	CMM6	CMM
Buffalo	Milk	BMM1	BMM
Buffalo	Milk	BMM2	BMM
Buffalo	Milk	BMM3	BMM
Buffalo	Milk	BMM4	BMM
Buffalo	Milk	BMM5	BMM
Buffalo	Milk	BMM6	BMM

## Data Availability

The original contributions presented in the study are included in the article/supplementary material, further inquiries can be directed to the corresponding author.

## Supplementary Materials

The following supporting information can be downloaded at: https://www.mdpi.com/article/10.3390/foods13091365/s1, Figure S1: Principal component analysis of the metabolites of four type milk samples; Figure S2: PLS-DA plots exhibiting discernible metabolic disparities among milk samples; Table S1: A total of 631 metabolites were identified and annotated; Table S2: Correlation coefficient between samples; Table S3: The results of K-Means cluster analysis; Table S4: The results of differential metabolite screening between GMMs and SMMs; Table S5: The results of differential metabolite screening between GMMs and CMMs; Table S6: The results of differential metabolite screening between GMMs and BMMs; Table S7: The results of differential metabolite screening between CMMs and BMMs; Table S8: The results of differential metabolite screening between CMMs and SMMs; Table S9: The results of differential metabolite screening between SMMs and BMMs.
