# Supplementary material for: Widely Targeted Metabolomic Analysis Revealed the Diversity in Milk from Goats, Sheep, Cows, and Buffaloes and Its Association with Flavor Profiles
Source: Foods. 2024 Apr 28;13(9):1365. doi: 10.3390/foods13091365 (PMC11083174; doi:10.3390/foods13091365)
Supplement: Supplementary file 1 [file foods-13-01365-s001.zip › supplementary_materials.pdf]

Supplementary Materials for

# Widely targeted metabolomics analysis identified characteristic flavor compounds in the milk from goats, sheep, cows, and buffaloes

Fuhong Zhang *et al.*

*Corresponding author. Email: luojun@nwafu.edu.cn

**This PDF file includes:**

Supplementary Text
Figures. S1 to S2
Tables. S1 to S9

## Supplementary Figures

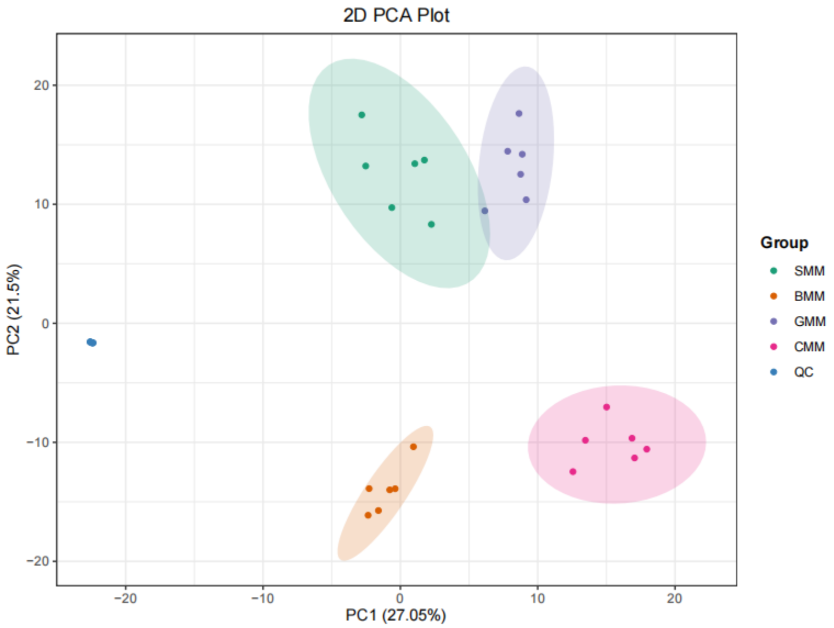


Figure S1. Principal component analysis of the metabolites of four type milk samples. Equal milk samples were mixed and used as quality control (QC).

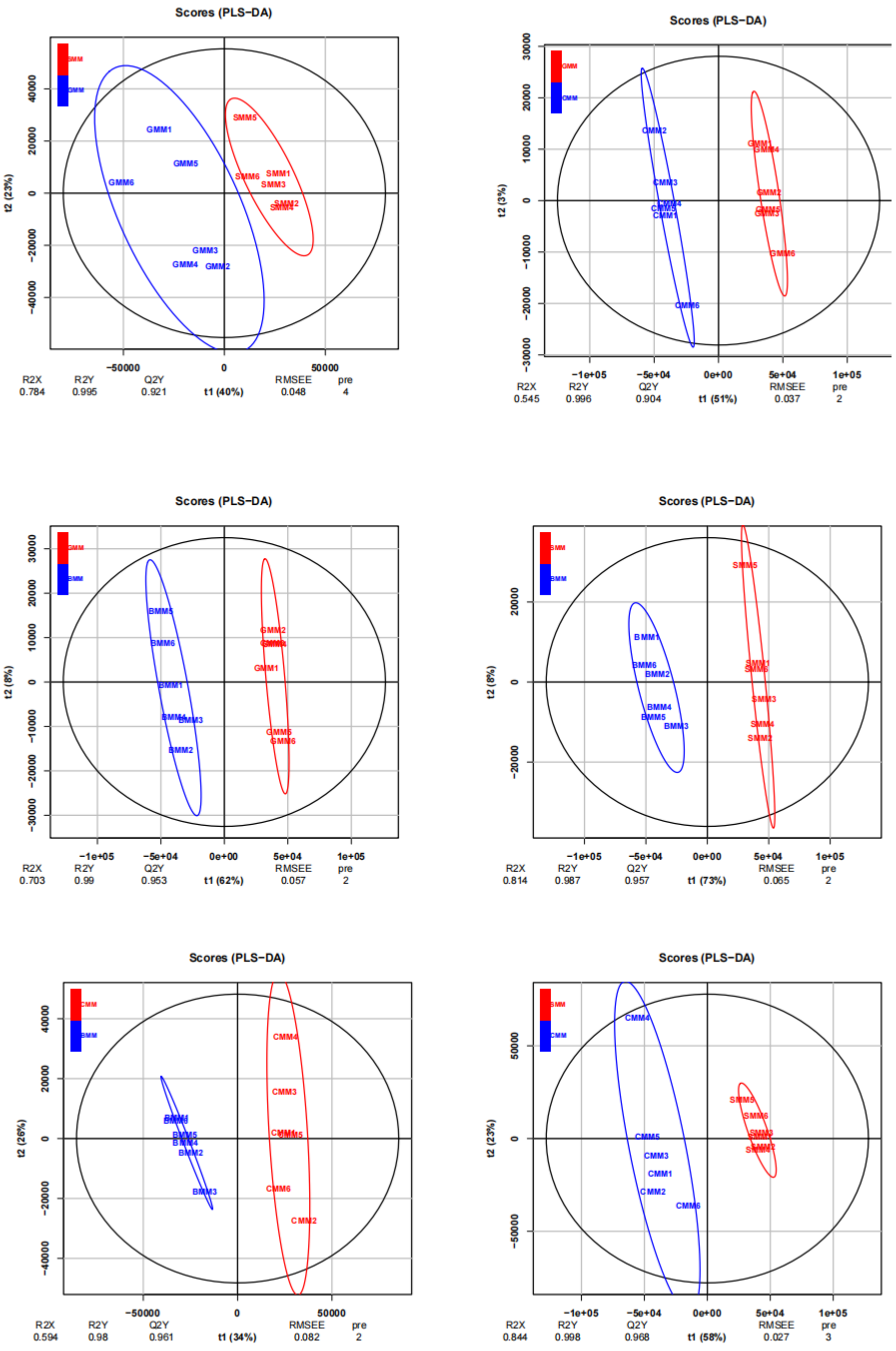


Figure S2. PLS-DA plots exhibiting discernible metabolic disparities among milk samples.

**Supplementary Tables**

**Table S1. A total of 631 metabolites were identified and annotated.**
(Excel table)
**Table S2. Correlation coefficient between samples.**
(Excel table)
**Table S3. The results of K-Means cluster analysis**.
(Excel table)
**Table S4. The results of differential metabolite screening between GMMs and SMMs.**
(Excel table)
**Table S5. The results of differential metabolite screening between GMMs and CMMs**.
(Excel table)
**Table S6. The results of differential metabolite screening between GMMs and BMMs.**
(Excel table)
**Table S7. The results of differential metabolite screening between CMMs and BMMs.**
(Excel table)
**Table S8. The results of differential metabolite screening between CMMs and SMMs**.
(Excel table)
**Table S9. The results of differential metabolite screening between SMMs and BMMs.**
